# From Paper to Digital: Performance and Challenges of the Electronic Hepatitis B Surveillance System in Ninh Binh, Northern Vietnam (2017–2022)

**DOI:** 10.3390/tropicalmed9120299

**Published:** 2024-12-05

**Authors:** Hien T. Nguyen, Thai Q. Pham, Duc M. Hoang, Quang D. Tran, Giang T. Chu, Thuong T. Nguyen, Nam H. Le, Huyen T. Nguyen, Khanh C. Nguyen, Florian Vogt

**Affiliations:** 1 National Centre for Epidemiology and Population Health, College of Health and Medicine, Australian National University, Canberra, ACT 2601, Australia; huyen.nguyen@anu.edu.au (H.T.N.); florian.vogt@anu.edu.au (F.V.); 2 Department of Communicable Disease Control, National Institute of Hygiene and Epidemiology, Hanoi 100000, Vietnam; pqt@nihe.org.vn (T.Q.P.); nck@nihe.org.vn (K.C.N.); 3 Research Methodology and Biostatistics Department, School of Preventive Medicine and Public Health, Hanoi Medical University, Hanoi 100000, Vietnam; 4 General Department of Preventive Medicine, Ministry of Health, Hanoi 100000, Vietnam; duchmvn@yahoo.com (D.M.H.); trandaiquang1984@gmail.com (Q.D.T.); 5 Ninh Binh Provincial General Hospital, Ninh Binh 430000, Vietnam; bschugiang@gmail.com (G.T.C.); thienthuong1986@gmail.com (T.T.N.); 6 Ninh Binh Center for Disease Control and Prevention, Ninh Binh 430000, Vietnam; lehoangnam06@gmail.com; 7 Field Epidemiology Training Program, National Institute of Hygiene and Epidemiology, Hanoi 100000, Vietnam; 8 The Kirby Institute, University of New South Wales, Sydney, NSW 2052, Australia

**Keywords:** hepatitis B, public health surveillance, simplicity, timeliness, data quality, acceptability

## Abstract

Hepatitis B remains a major public health issue in Vietnam. Mandatory reporting to the national electronic communicable disease surveillance system (eCDS) has been required since July 2016. We conducted an evaluation of the hepatitis B surveillance system in Ninh Binh, the province with the highest reported burden of hepatitis B in Northern Vietnam, between 2017 and 2022. Using the CDC’s guidelines for evaluating public health surveillance systems, we assessed four key attributes: simplicity, timeliness, data quality, and acceptability. This retrospective evaluation included document reviews, analysis of hepatitis B data, and in-depth interviews with provincial-level healthcare staff involved in the reporting of hepatitis B cases. The results showed that the eCDS improved reporting frequency, provided more detailed case information, and enhanced data accessibility compared to the previous paper-based system. However, the system faced several challenges, including unclear objectives, difficulties in distinguishing acute from chronic cases, insufficient training for staff, lack of supervision for data quality, and technical software issues. Despite these challenges, stakeholders found the system acceptable but emphasized the need for improvements, including revising the system’s objectives, automating case classification, enhancing training, securing funding for maintenance, and implementing regular data review processes.

^‡^ These authors contributed equally to this work.

## 1. Introduction

Hepatitis B, the cause of approximately 254 million chronic infections around the world with 1.1 million deaths in 2022, remains a major health challenge globally with the highest burden of this disease observed in the Western Pacific and African regions [1]. To address the global impact of viral hepatitis including hepatitis B, the World Health Organization (WHO) has unveiled its worldwide health sector strategy for 2022–2030 with the goal of ending hepatitis B infections by 2030 [2]. During 2020–2022, the COVID-19 pandemic was found to have a negative impact on the routine surveillance of hepatitis B in some countries in the world [3,4].

Located in Southeast Asia with a population of nearly 100 million, Vietnam is among the ten countries that collectively account for nearly two-thirds of the global burden of viral hepatitis B and C, highlighting its significant public health challenge. According to the Global Hepatitis Report 2024 by WHO, Vietnam recorded 6.5 million hepatitis B infections across all age groups in 2022, representing 2.4% of the global burden [5].

Hepatitis B can be prevented through vaccination. The HBV vaccine was introduced worldwide in 1981 and has shown an efficacy of 80–100% in preventing the disease [6]. The WHO advises that all infants should receive the HBV vaccine ideally within the first 24 h after birth, followed by 2 or 3 additional doses at least 4 weeks apart to complete the vaccination regimen [7]. While Vietnam has started implementing subsidized HBV vaccination for infants since 2003, there is no equivalent program for adults, particularly those born before the early 2000s [8]. Consequently, only high-risk groups are prioritized for vaccination, as recommended in the National Action Plan (NAP) for Viral Hepatitis Prevention and Control 2021–2025 [9], leaving many healthy adults unprotected and vulnerable to HBV, especially in a country with high endemicity like Vietnam, with only 41.7% of infected cases being diagnosed and 1.4% of diagnosed cases being treated in 2022 [5].

In Vietnam, hepatitis B cases in all provinces are regularly reported through an indicator-based surveillance system. With the issuance of Circular 54/2015 by the Ministry of Health (MOH) on Guidelines for Reporting and Declaring Infectious Diseases and Outbreaks, starting from 1 July 2016, the national electronic communicable disease surveillance system (eCDS) was officially launched in Vietnam and has become the primary channel for reporting notifiable infectious diseases, including for hepatitis B [10]. According to the MOH’s guideline, each case of this disease must be reported within 48 h of clinical diagnosis. Compared to the previous paper-based surveillance system, the new online system is expected to quickly aggregate data on infectious disease cases, contributing to the early detection of outbreaks and reducing data duplication between reporting units at different levels [9,10].

Ninh Binh, located in the south of the Northern Delta, is the province that recorded the highest incidence of hepatitis B infections per 100,000 population in North Vietnam during 2017–2022 in the eCDS. Given the high burden of hepatitis B in Ninh Binh, we chose to evaluate the hepatitis B surveillance system in this province through four key attributes including simplicity, timeliness, data quality, and acceptability. Results of this evaluation may contribute to the optimization of the system’s performance and broader public health actions. We expect our findings will inform Ninh Binh province in particular, and in Vietnam in general, in adjusting the surveillance strategy towards the WHO’s aim to eradicate viral hepatitis as a significant public health concern by the year 2030.

## 2. Materials and Methods

### 2.1. Study Design

We evaluated the hepatitis-B-indicator-based surveillance system in Ninh Binh province, Vietnam, over the period of six years from 2017 to 2022. This study followed the US Centers for Disease Control and Prevention (US CDC, Atlanta, GA, USA) Guidelines for Evaluating Public Health Surveillance Systems [11], and assessed four attributes of the surveillance system: simplicity, timeliness, data quality, and acceptability. To evaluate simplicity, timeliness, and acceptability, we utilized a combination of desk reviews and stakeholder interviews to gather relevant information. To assess data quality, we applied a mixed-methods explanatory sequential design approach. In the first phase, secondary quantitative data extracted from the eCDS were analyzed to identify patterns and trends in hepatitis B cases. The second phase involved conducting qualitative interviews with relevant staff to provide deeper insights and contextualize the findings from the quantitative analysis.

### 2.2. Setting

Ninh Binh is located in the southernmost part of the Red River Delta in Vietnam, covers 1411.8 square kilometers [12], and has a population of approximately 1.02 million people [13]. Ninh Binh is subdivided into eight district-level units, including two municipal cities and six districts. The province’s health system is led by the provincial Department of Health and includes several provincial and district-level hospitals, health centers, and commune health stations [14].

### 2.3. Case Definition

According to the guidelines from Vietnam’s MOH [15], the definitions of confirmed cases for acute and chronic hepatitis B are as follows:

Acute hepatitis B: a case that has both of the following criteria:

- Immunoglobulin M antibody to hepatitis B core antigen (anti-HBc IgM)-positive;
- Hepatitis B surface antigen (HBsAg)-positive (or -negative in the window period).

Chronic hepatitis B: a case that meets both of the following criteria:

- HbsAg-positive for more than 6 months, or HBsAg-positive and anti-HBc IgM-positive;
- There is evidence of progressive histopathological damage or cirrhosis without other causes.

### 2.4. Data Collection

For the quantitative data, we extracted data for all hepatitis B cases in Ninh Binh province recorded in the eCDS from 1 January 2017 to 31 December 2022. We imported these data into a Microsoft Excel software (Redmond, WA, USA) and performed descriptive analyses using the Pivot Table function to summarize and interpret the data.

For the qualitative component, we conducted a desk review and developed an in-depth interview guideline based on the Vietnamese government’s regulations of operating notifiable disease surveillance systems [10] and guidelines from the US CDC on evaluating specified attributes of disease surveillance systems [11]. Regarding the selection of interviewees, we intentionally selected three staff from Ninh Binh Provincial General Hospital (PGH) because this hospital reported 96.9% of all hepatitis B cases in the eCDS system in Ninh Binh province over the period of 2017–2022. The participants included one medical doctor and one nurse from the Department of Communicable Diseases, as well as the Head of the Department of General Planning. These individuals were selected based on their active involvement in the hepatitis B reporting system in the province. The interviews were conducted in-person and audio-recorded with verbal consent from the interviewees, in compliance with an ethical waiver obtained from the Australian National University Human Research Ethics Committee under the protocol number 2017/909. Respondents were asked to describe their roles in hepatitis B surveillance, provide their insights about the four selected attributes of this system, and give suggested recommendations.

### 2.5. Definitions of System Attributes

#### 2.5.1. Simplicity

To evaluate the simplicity of this surveillance system, we assessed the data flow including levels of reporting, reporting structure, and data management based on system documentation and feedback from staff of Ninh Binh Provincial General Hospital (PGH). The system’s simplicity can be assessed based on certain criteria. For instance, if the case definition is easy to apply and the person identifying the case is also responsible for analyzing and using the information, the system is considered simple. Conversely, a more complex system may involve additional steps such as special laboratory tests for confirmation, detailed case investigations including home visits, multiple levels of reporting, and the need for specialized training to collect or interpret data [11].

#### 2.5.2. Timeliness

In our study, the period that elapsed between the hepatitis B diagnosis by a physician or laboratory and the date reported in the eCDS was used as an indicator to assess the timeliness of the hepatitis B surveillance system. Given that the aforementioned information was not available on the eCDS or other systems, we mainly described this attribute based on the views from health staff from Ninh Binh PGH who were involved in the reporting system.

#### 2.5.3. Data Quality

For data quality, we assessed the consistency between quantitative data and responses from staff at Ninh Binh PGH. We also evaluated the completeness of variables including vaccination status, epidemiological history, date of onset, date of sampling, date of testing result, current situation (treatment results for inpatients), and date of hospital discharge/referral/death. To calculate the percentage of data completeness, we divided the number of complete records by the total number of records for each variable.

#### 2.5.4. Acceptability

Regarding acceptability, we depicted the readiness of individuals and organizations to engage in the surveillance system from opinions of staff at Ninh Binh PGH.

### 2.6. Data Analysis

To calculate the incidence of hepatitis B in Ninh Binh and other provinces in Northern Vietnam during the 2017–2022 period, we divided the number of new hepatitis B cases reported each year in the eCDS by the total provincial population, which was obtained from the 2019 Vietnam Population and Housing Census [16]. To describe the system’s operation, we integrated relevant content from government documents and responses from interviewees. Descriptive analysis was conducted using the Pivot Table function in Microsoft Excel to assess the quality of surveillance data. The qualitative data from interviews were analyzed using a thematic analysis approach. Themes were identified inductively, allowing patterns to emerge directly from the data. Verbatim quotes were chosen to illustrate key themes, with selection criteria focusing on their representativeness of broader participant perspectives.

## 3. Results

### 3.1. Epidemiology of Hepatitis B in Ninh Binh Province, 2017–2022

**Figure 1:**
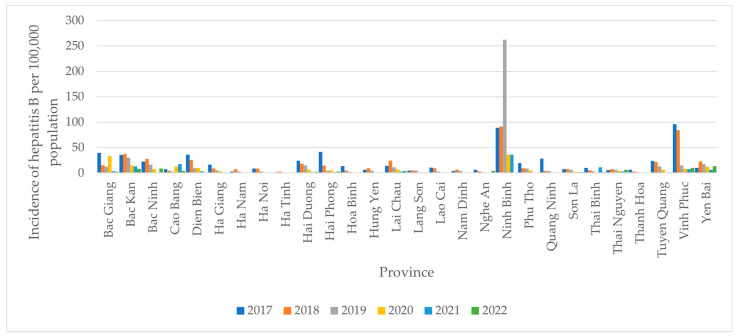
Incidence of hepatitis B per 100,000 population-years in 28 northern provinces in Vietnam 2017–2022.

Figure 1 shows the incidence of hepatitis B per 100,000 population among 28 provinces in Northern Vietnam over the six years of 2017–2022. Among these provinces, Ninh Binh stood out as the province with the highest incidence of hepatitis B during this period, with a peak in 2019 of 262.6 cases per 100,000 population. The unusually high number of reported hepatitis B cases (2579) in Ninh Binh province in 2019 was largely attributed to intensified community screening initiatives. The PGH began a hepatitis B screening program in 2016, scaling up efforts in 2018–2019 with bi-monthly screenings across various communes. This led to the early detection of suspected cases and prompted patients to register for local care due to its affordability and convenience compared to central-level hospitals. However, community screenings were disrupted during the COVID-19 pandemic, causing a drop in reported cases in subsequent years. Efforts to resume screenings began in 2022.

*“To detect hepatitis B cases early in the community, our hospital launched a screening program in 2016. In 2018–2019, we increased screening activities in various communes, conducting them twice a month. From these efforts, we identified several suspected hepatitis B cases and referred them to our hospital for further diagnosis and treatment. After learning about the hepatitis B management program at the provincial hospital, patients registered immediately, as it saves considerable travel and treatment costs compared to going to central-level hospitals. In 2020–2021, due to the COVID-19 pandemic, we were unable to conduct community screenings, and patients rarely visited the hospital due to travel restrictions. As a result, the number of new hepatitis B cases reported to the eCDS decreased. Since 2022, we have been working to resume community screenings. This is the main reason the number of reported hepatitis B cases in 2019 was exceptionally high compared to other years”*.

(Interviewee 1)

Table 1 shows characteristics of all 5066 hepatitis B cases in Ninh Binh province reported in the eCDS over 6 years from 2017 to 2022 with a median age of 46 years (interquartile range, 34–58 years). Most of the cases were male (60.2%), farmer by profession (51%), and unvaccinated against HBV (68%). A total of 4908 cases (96.9%) were reported by Ninh Binh PGH in the surveillance system, with the remaining 3.1% reported by national hospitals, other provincial hospitals, district hospitals, and commune health stations.

### 3.2. Objectives of the Surveillance System

The objectives of the surveillance system were not clearly defined in the Ministry of Health Guidelines for Reporting and Declaring Infectious Diseases and Outbreaks in Vietnam (Circular 54/2015). The Guidelines only mentioned types of reporting including case reports, weekly reports, monthly reports, annual reports, outbreak reports, and extraordinary reports [10].

### 3.3. Simplicity

**Figure 2:**
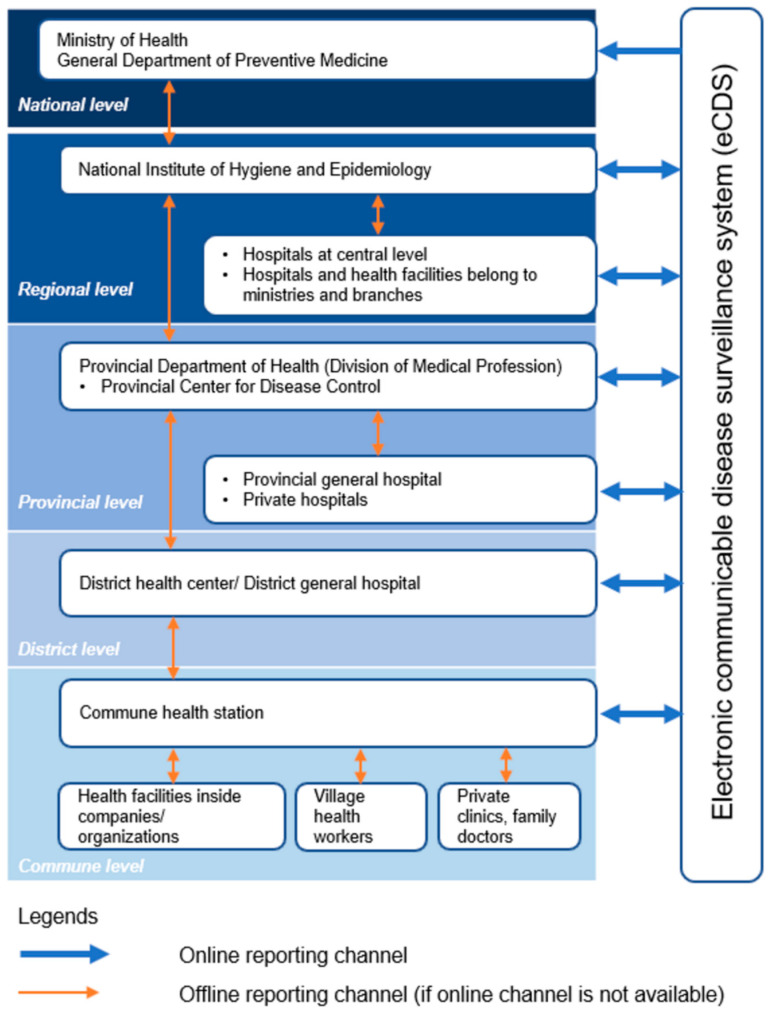
Hierarchical structure and reporting flow of Ninh Binh’s hepatitis B surveillance system, including both online and offline reporting channels across all administrative levels.

The hepatitis-B-indicator-based surveillance system is part of the national notifiable disease surveillance system in Vietnam. Figure 2 shows the data flow in the hepatitis B surveillance system in Ninh Binh province. It consists of multiple levels of reporting. At the commune level, a suspected case may be detected by healthcare workers at health facilities inside companies or organizations, by village health workers, by healthcare workers at private clinics, or by family doctors. The healthcare worker or doctor who identifies the suspected hepatitis B case must notify the commune health station (CHS) of this person for further investigation and verification. If the case is then confirmed as a positive hepatitis B case, the CHS should report it to the eCDS within 48 h from the time of diagnosis confirmation. District health centers, district general hospitals, and provincial hospitals can also directly access the eCDS to report hepatitis B cases that they identified.

The CHS, district health center/district hospital, or provincial hospitals can monitor other cases in this province reported by other units. In case online reporting to the eCDS is impossible, health facilities should report to higher levels via an official letter (paper-based), fax, or e-mail. For emergency cases, health staff can report to higher authorities in person or by phone call first, and then report to the eCDS or provide a written report within 24 h. The two-way arrows in this system mean one way of data entry to the system and one way for other organizations to verify and send feedback about these cases.

In Ninh Binh PGH, the Department of Communicable Disease has staff designated as focal points who report all cases of hepatitis B managed by this hospital to the eCDS. In terms of simplicity, the staff reflected during interviews that it did not take too much time or effort to enter the information of a hepatitis case into the eCDS. In comparison, the previously reported paper-based system required them to report monthly, quarterly, and yearly to higher levels, while the eCDS only requires them to report case data once because health authorities in lower levels and higher levels can access these data.

However, this system requires follow-up actions by health staff for hepatitis B inpatients. After entering the patients’ data at the time of hospital admission, nurses must monitor their treatment outcomes and then update the case’s data to the eCDS when patients are discharged from the hospital, or referred to other medical facilities, or die.

In addition, respondents were unsure of the eCDS reporting requirements. For example, chronic hepatitis B patients need to go to outpatient clinics inside Ninh Binh PGH every month until the end of their lives to obtain medicines covered by health insurance. Health staff at Ninh Binh PGH were unsure if they only needed to report new cases of hepatitis B, or if they must also update the system about every return visit of outpatients. In the past, some cases were reported more than once into the eCDS based on re-visits, and health staff felt it was complicated and time-consuming to report like this. They also faced significant challenges in reporting hepatitis B cases due to time constraints from managing approximately 4000 cases annually and handling additional administrative demands, such as maintaining medical records for all outpatients.

*“Only the first patient visit should be reported to the surveillance system, not their follow-up visits. As nurses, we have various responsibilities, such as preparing health records and assisting doctors and patients, so we do not have much time for reporting. Every year, we manage about 4000 cases of hepatitis B, with many outpatients visiting our clinics monthly. Unlike other health conditions, hepatitis B is a special case, requiring us to prepare and store the medical records of all outpatients coming to our hospital”*.

(Interviewee 2)

In the early stages of implementing this new surveillance system, there was only one training provided by Ninh Binh CDC in 2016, and only one staff member from the Department of General Planning at Ninh Binh PGH was sent to this training. On completion of this training, he came back and guided health staff of the Department of Communicable Disease to work with the eCDS, but his verbal instructions were unclear, and no training materials were provided to health staff who directly work with this system. Whenever questions or concerns arose, the Chief Nurse from Department of Communicable Disease phoned a focal point from Ninh Binh CDC to receive verbal guidance without any supporting documents.

According to our evaluation criteria for simplicity, this surveillance system appears to be complex, not simple. This complexity arises from the necessity for health staff to conduct follow-up actions to update laboratory test results and patient outcomes, as well as the requirement for specialized training to collect and interpret data.

### 3.4. Timeliness

According to guidelines from Vietnam’s MOH, hepatitis B is a ‘Class B’-reportable infectious disease, which must be reported within 48 h from the time of confirmed diagnosis [10]. However, quantitative data extracted from the eCDS did not include information of the reporting date or date of confirmed diagnosis for each hepatitis B case. For time variables, we can only view the date of sampling, date of health check/hospitalization, and date of hospital discharge/referral/death. Therefore, we were unable to assess the timeliness of this surveillance system through eCDS data.

When asked about the duration from the time of diagnosis to the time of reporting a hepatitis B case to the eCDS, health staff from the Department of Communicable Disease in Ninh Binh PGH reported that they normally input data into the eCDS within a day.

*“Almost all hepatitis B cases with positive diagnoses are reported the same day. In our department, all staff agree and follow the practice of reporting infectious disease cases to eCDS in the late afternoon each day”*.

(Interviewee 1)

However, in some situations when the eCDS had technical problems that made data entry impossible, there were some delays in reporting hepatitis B cases.

*“In the early stage of working with eCDS, system occurred quite frequently, which prevented us from updating hepatitis B cases in the software, leading to some cases being reported late. Recently, the system has been running more smoothly”*.

(Interviewee 2)

The COVID-19 pandemic also negatively impacted on the timeliness of reporting. During peak times of SARS-CoV-2 transmission from 2020 to 2022 in this locality, health staff of the Department of Communicable Disease were extremely busy implementing measures to prevent COVID-19 outbreaks and treating severe cases; therefore, they did not have time to report in the eCDS in a timely manner.

*“During the peak of the COVID-19 pandemic, we were working hard to combat this disease, so we did not have time to report other cases to eCDS promptly”*.

(Interviewee 2)

### 3.5. Data Quality

#### 3.5.1. Variables in the Reporting System

**Figure 3:**
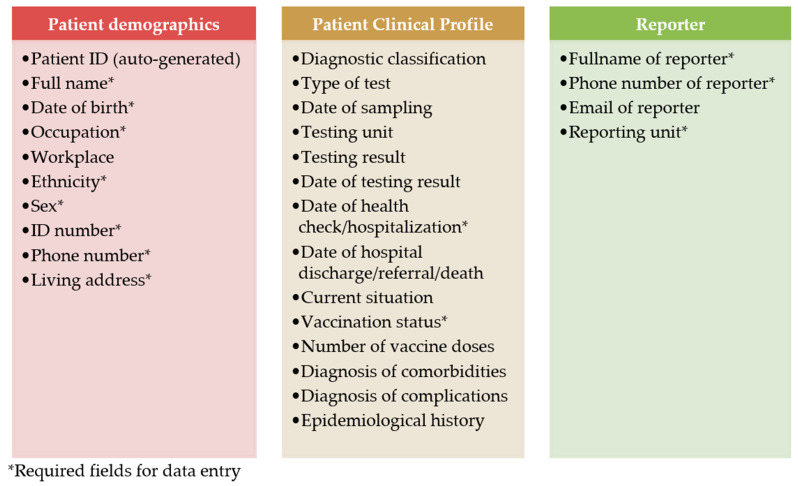
All variables in the eCDS reporting system of hepatitis B, categorized into patient demographics, patient clinical profile, and reporter’s information.

Figure 3 lists all variables that appear on the eCDS when reporting a hepatitis B case. There are three main categories of information: patient demographics; patient’s clinical profile; and reporter. There are 13 required (those marked with asterisk) and 15 optional fields in a case report form.

#### 3.5.2. Data Consistency

Health staff from the Department of Communicable Diseases in Ninh Binh PGH stated that they only reported hepatitis B cases confirmed by positive HBsAg test results from their laboratory or central-level hospitals.

*“For hepatitis B, we only reported cases that had positive HBsAg test results provided by our laboratory or another hospital at central level”* .

(Interviewee 1)

However, this claim was inconsistent with data observed in the eCDS, suggesting potential discrepancies in reporting practices. For diagnostic classification, there were 4877 laboratory-confirmed cases (96.3%), 179 (3.5%) were suspected by clinical diagnosis, and 10 (0.2%) were probable cases. For testing results, there were two cases who were reported as negative in 2018 and 2021, and 372 (7.3%) with “result pending”. Forty-eight cases (1%) were reported as not involving any hepatitis B tests. The ways of reporting testing types were not consistent between different cases and not compatible with the hepatitis B case definitions as per recommendations by Vietnam’s MOH (Table 2).

#### 3.5.3. Data Completeness

Among the seven variables that were assessed for data completeness, there were one mandatory field (vaccination status) and six optional fields. Normally, all required fields have a data completeness of 100%. However, for vaccination status, 32% were “Unknown”; therefore, we also included this variable for data completeness concerns.

As shown in Table 3, “Date of sampling” was the variable with the highest data completeness (98.7%), and the data completeness of “date of onset”, “current situation” (only selected treatment results for inpatients), and “date of hospital discharge/referral/death” were 43.5%, 63.5%, and 77.1%, respectively. There were no data for “epidemiological history” and “date of testing result”. Health staff from Ninh Binh PGH reported that a lack of knowledge among patients about how they contracted hepatitis B made it difficult to complete the “Epidemiological history” field in the surveillance system. This gap in patient information led to missing data in the eCDS.

*“Most patients did not know how they contracted HBV, so we had no data to report in the “Epidemiological history” field”*.

(Interviewee 2)

### 3.6. Acceptability

In general, all respondents from Ninh Binh PGH viewed the current surveillance system as acceptable for monitoring hepatitis B cases in their locality. Compared to the previous paper-based surveillance system, the eCDS has several advantages in terms of reporting frequency, data quality, and accessibility (Table 4).

Prior to 2016, staff from the Department of General Planning aggregated data only based on the number of infections and deaths, and then submitted paper-based reports to higher levels monthly, quarterly, and annually. In that old surveillance system, only authorities in higher levels could access the data reported by lower levels. In contrast, in the eCDS, reporters only need to provide input data once for each case without aggregating data for month, quarter, or year. More variables included in case report forms and warning functions of the software help in reducing duplications in reporting.

*“Our previous reporting system for hepatitis B was paper-based. At that time, the Department of General Planning was responsible for aggregating data on all notifiable diseases managed by this hospital and submitting reports to the provincial CDC and Department of Health on a monthly, quarterly, and annually basis. Now, the Department of Communicable Disease oversees reporting case information through the new computer-based system, so we no longer need to prepare regular reports. We only enter case data once, and other health authorities at different levels can directly access this information via eCDS”*.

(Interviewee 3)

If health staff want to check the medical history of a patient, they can easily and instantly search in the eCDS, which is more convenient and timelier than finding the patient’s information from paper health records. One additional strength is that health facilities at lower levels, such as CHS and the district health center, can access all data for all cases in this province; therefore, they can easily find patients living in their areas and verify patient data.

*“With the old surveillance system, we only reported the number of infections and deaths. The new electronic reporting system provides more detailed data on each case. If we need patient information, we can simply look it up on eCDS—it is much faster and more convenient than the paper-based system. It also reduces data duplication, as the system warns us if we enter the same information from previously reported cases. This software generally helps both preventive and clinical units access data more quickly and easily”*.

(Interviewee 2)

In addition, one staff member from the Department of Communicable Disease made the point that they do not have any recommendations for the design of the current surveillance system, because its objectives were not clear to them.

*“To optimize the design of a surveillance system, its objectives should be clearly communicated to all stakeholders. This way, everyone can understand the importance of this system and work towards providing the most accurate and timely data to eCDS. Currently, the objectives of this system are unclear to us, so we are unable to offer recommendations for its design and operation”*.

(Interviewee 1)

## 4. Discussion

Our evaluation provided a foundational understanding on some strengths and weaknesses of the hepatitis B surveillance system in Ninh Binh province over 6 years from 2017 to 2022 and generated several recommendations for optimizing its operation and usefulness.

The major strengths of the current hepatitis B surveillance system in comparison with the previous paper-based version include reduced reporting frequencies, the provision of more detail of each case, less duplication of data, and improved data accessibility for health authorities at diverse levels.

In the age of digital technology, using an electronic system to manage surveillance data enables users to quickly identify disease patterns, populations at high risk, and potential outbreaks in the locality. A number of developed and developing countries also operate electronic reporting systems for fast communication of hepatitis B cases including Germany [17], Italy [18], and China [19]. In contrast, Albania is one example of a country that still manages a paper-based surveillance system for hepatitis B, and the evaluation of hepatitis B and C surveillance in this country concluded that a web-based reporting system is required to improve usefulness, data quality, and efficiency of this system [20].

Despite the above-mentioned assets, there are various challenges that need to be addressed to improve the performance of this hepatitis B surveillance system. First and foremost, the lack of clearly defined objectives of the surveillance system in the Ministry of Health guidelines poses a significant challenge in assessing the surveillance system’s usefulness. It is imperative for the Ministry of Health of Vietnam to thoroughly review and redefine the objectives of the eCDS to ensure clarity and alignment with its intended purpose.

One of the most critical criteria for evaluating the quality of a disease surveillance system is whether it accurately reflects the patients who meet the standard case definition. The surveillance systems in other countries clearly classify the reporting patients into two clinical types of hepatitis B cases—acute and chronic conditions [17,18]. Our findings revealed that the current hepatitis B surveillance system in Vietnam is unable to differentiate between acute and chronic hepatitis B cases as per guidelines by Vietnam’s MOH. A similar issue was reported in a study from China where the country’s hepatitis B surveillance system also failed to distinguish between acute and chronic infections [19]. This issue could be resolved by adjusting the classification of testing types in the eCDS to meet the criteria of standard case definitions for acute and chronic patients. In addition, it may be more efficient if an algorithm is installed in the software to review the input data and automatically classify acute and chronic hepatitis B cases. This function could not only reduce the workload of reporting individuals, but also provide quick advice for physicians to diagnose the disease conditions more accurately, thereby giving the appropriate treatments for patients. Our country should learn from Germany’s experience on this, since they already adapted this technology to their surveillance system [17].

Although health staff from Ninh Binh PGH had been working with the current hepatitis B surveillance system for more than six years, they were still unclear about its objectives and uncertain about the data that should be reported in the eCDS. This problem can lead to delayed reporting, time wasting, and extra workload for health workers, and may result in lower acceptability of the system. One of the main reasons for this confusion was the lack of regular trainings and clear guidelines for people involved with this system. To address this uncertainty, training should be organized and instruction documents should be distributed to all users.

Timeliness is a crucial attribute for the effectiveness of a surveillance system, typically measured by the time interval between a hepatitis B-confirmed diagnosis by a physician or laboratory and its reporting to the notification system, as highlighted in studies from other countries [21,22,23]. However, the current hepatitis B surveillance system in Ninh Binh does not track this critical period, leaving a gap in the assessment of reporting timeliness. To improve the system, it is essential to include fields for both the date of confirmed diagnosis and the date of reporting. This addition would enable the verification of timeliness, ensuring alignment with the MOH’s guidelines and enhancing the overall efficiency of the surveillance system.

Data inconsistency and incompleteness were persistent issues in hepatitis B reporting in Ninh Binh province from 2017 to 2022, including important information such as vaccination status and epidemiological history. Similarly, a study examining hepatitis B data across European countries from 2006 to 2012 found that HBV vaccination and transmission route data were also poorly reported [24]. To address this issue, the Vietnam MOH should establish specific authority and mechanisms for regularly reviewing and providing feedback on data reported in the eCDS. The surveillance results should also be analyzed in depth and disseminated to all relevant stakeholders on a regular basis to increase the usefulness of this system. These recommendations were also suggested by other studies in Italy [18] and Iran [25].

In our evaluation, we found that the “epidemiological history” field was blank for all records of hepatitis B. While it is understandable that patients rarely know how they contracted HBV, hospital staff reported that there is currently no questionnaire to collect risk factor data for HBV in Vietnam. Therefore, a case investigation questionnaire should be developed for health professionals to interview patients and capture potential risk factor data. This would help understand trends in risk factors and enable the completion of the “epidemiological history” field. This is also recommended in the guidelines of the US CDC [26] and Public Health England [27].

Technical errors and bugs are inevitable problems for every piece of software; therefore, regular monitoring and maintenance are required to handle these issues promptly to minimize the number of delayed reports. However, the eCDS was developed and sponsored by Viettel, a state-owned enterprise managed by the Vietnam Ministry of National Defence. Sometimes, when software glitches occurred, the responsible authority from the MOH had to contact a focal point of Viettel to ask for their support, which can be time-consuming and inconvenient. It would be more effective if the MOH could allocate some funds to own this software and directly manage and maintain the operation of the eCDS.

In this study, we found that the COVID-19 pandemic negatively affected routine reporting of hepatitis B cases in Ninh Binh province as health workers had to devote all their efforts and time to prevent SARS-CoV-2 transmission and treat severe cases. This result is consistent with previous findings from other research in the UK [4] and in some European countries [3]. This issue calls for action to link and synchronize the eCDS with other existing software such as hospital management and the national vaccination database to automatically update the case information and reduce manual work for the health workforce, especially in crises like the COVID-19 pandemic.

Based on the evaluation findings, several key recommendations were made to enhance the hepatitis B electronic surveillance system in Ninh Binh, Vietnam. Firstly, the MOH should review and clearly define the objectives of the eCDS to ensure alignment with its intended purpose. Software enhancements, including installing an algorithm to classify acute and chronic hepatitis B cases automatically, are essential for improving data accuracy. The MOH should also allocate funds for ongoing software maintenance to address technical issues promptly. To reduce the reporting burden on health staff, linking and synchronizing the eCDS with other systems, such as hospital management and national vaccination databases, is recommended. Regular training sessions and detailed guidelines should be provided to all individuals involved in eCDS operations to enhance capacity. Additionally, a designated authority should be assigned to supervise and regularly review reported data to ensure consistency and completeness. A local public health dashboard should be developed to enhance data visualization, improve accessibility for stakeholders, and facilitate the timely identification and resolution of data quality issues. Surveillance data should be thoroughly analyzed and disseminated to relevant stakeholders to enhance the system’s usefulness. Lastly, organizing workshops for experience sharing among eCDS users across different provinces will foster discussions on system improvements and encourage the adoption of best practices.

Due to limited time, data availability, and resources, we acknowledge various limitations of this evaluation. Firstly, some crucial attributes of the surveillance system such as representativeness, sensitivity, and stability were not investigated or discussed. Secondly, we only interviewed three health staff members from Ninh Binh PGH as they were the primary contributors to the reporting system, accounting for 96.9% of the cases reported in the eCDS. We did not include all types of stakeholders across the system at lower and higher levels and at other health facilities in Ninh Binh province. These stakeholders may have different views and experiences when working with the eCDS and may provide further recommendations to improve the current system. Thirdly, the research team focused solely on analyzing secondary quantitative data extracted from the eCDS to assess the quality of the surveillance data. We did not utilize any quantitative tools to measure the respondent’s feedback on other attributes of the surveillance system, which may have limited the depth and scope of this evaluation. Fourth, there may be potential biases related to the underreporting or overreporting of cases within this surveillance system. Due to the lack of corroborative data from other sources, we cannot ascertain the presence, magnitude, or impact of these biases on the results. Lastly, our study provided insights from health workers in only one province in Northern Vietnam; thus, the findings may not be generalized for other areas that have different settings. Further comprehensive evaluation should be conducted on a larger scale in the future to determine the best practices and key challenges of the hepatitis B surveillance system across the country and to provide viable solutions for improvements across the system.

## 5. Conclusions

To summarize, the hepatitis B surveillance system in Ninh Binh province, Vietnam, was acceptable for monitoring this disease pattern in the locality during 2017–2022. Certain concerns such as the lack of frequent trainings, the inability of the system to distinguish acute and chronic hepatitis B cases, the inability for the early detection of poor data quality, and technical issues reduced its performance. In light of the findings, several recommendations were made for the system enhancement, including (1) a thorough review and redefinition of eCDS objectives by the Ministry of Health, (2) installing algorithms in the software to automatically classify hepatitis B cases, (3) allocating funds for software maintenance to ensure smooth operation, (4) linking the surveillance system with other software for streamlined reporting, (5) conducting regular training on system objectives and operations, (6) providing detailed guidelines for system usage, (7) establishing a mechanism for regular data review to ensure consistency and completeness, (8) analyzing and disseminating surveillance data to relevant stakeholders, and (9) organizing workshops for experience sharing and system improvement discussions among users from different provinces.

## Figures and Tables

**Table 1:** Characteristics of hepatitis B cases in Ninh Binh province, 2017–2022 (n = 5066).

Characteristics	Number of Patients (n = 5066)	Percentage (%)
Age (Median, IQR)	46 (34–58)	
Age group		
<18	28	0.6%
18–29	733	14.5%
30–39	1138	22.5%
40–49	1035	20.4%
50–59	1024	20.2%
≥60	1108	21.9%
Sex		
Male	3050	60.2%
Female	2016	39.8%
Occupation		
Farmer	2586	51%
Blue-collar worker	645	12.7%
Retiree	498	9.8%
White-collar worker	407	8%
Student	93	1.8%
Armed forces	59	1.2%
Other	778	15.4%
Vaccination status		
Unvaccinated	3445	68%
Unknown	1621	32%
Reporting unit		
Ninh Binh PGH	4908	96.9%
Others	158	3.1%

**Table 2:** Specific variables for data consistency concerns.

Variables	2017 n (%)	2018 n (%)	2019 n (%)	2020 n (%)	2021 n (%)	2022 n (%)	Total n (%)
Diagnostic classification							
Laboratory-confirmed	871 (99.7%)	884 (98.4%)	2573 (99.7%)	268 (75.3%)	279 (79%)	2 (40%)	4877 (96.3%)
Suspected	2 (0.2%)	10 (1.1%)	4 (0.2%)	87 (24.4%)	73 (20.7%)	3 (60%)	179 (3.5%)
Clinical	1 (0.1%%)	4 (0.5%)	3 (0.1%)	1 (0.3%)	1 (0.3%)	0	10 (0.2%)
Testing results							
Positive	862 (98.6%)	810 (90.2%)	2356 (91.3%)	319 (89.6%)	293 (83%)	4 (80%)	4644 (91.7%)
Negative	0	1 (0.1%)	0	0	1 (0.3%)	0	2 (0%)
Result pending	11 (1.3%)	87 (9.7%)	223 (8.6%)	15 (4.2%)	35 (9.9%)	1 (20%)	372 (7.3%)
No test	1 (0.1%)	0	1 (0%)	22 (6.2%)	24 (6.8%)	0	48 (1%)
Type of test							
Rapid test	846 (96.8%)	865 (96.3%)	2566 (99.5%)	309 (86.8%)	239 (67.7%)	4 (80%)	4829 (95.3%)
Mac-Elisa	19 (2.2%)	31 (3.5%)	8 (0.3%)	18 (5.1%)	67 (19%)	1 (20%)	144 (2.8%)
PCR	6 (0.7%)	0	4 (0.2%)	1 (0.3%)	2 (0.6%)	0	13 (0.3%)
HbsAg	1 (0.1%)	0	1 (0%)	2 (0.6%)	1 (0.3%)	0	5 (0.1%)
HBV DNA	0	2 (0.2%)	0	1 (0.3%)	0	0	3 (0.1%)
Blood count	1 (0.1%)	0	0	0	0	0	1 (0%)
Unknown	0	0	0	3 (0.8%)	20 (5.7%)	0	23 (0.5%)
No test	1 (0.1%)	0	1 (0%)	22 (6.2%)	24 (6.8%)	0	48 (1%)

**Table 3:** Specific variables for data completeness concerns.

Variable	2017 n (%)	2018 n (%)	2019 n (%)	2020 n (%)	2021 n (%)	2022 n (%)	Total n (%)
Vaccination status	642 (73.5%)	517 (57.6%)	1625 (63%)	304 (85.4%)	352 (99.8%)	5 (100%)	3445 (68%)
Date of onset	412 (47.1%)	300 (33.4%)	806 (31.2%)	336 (94.4%)	346 (98%)	4 (80%)	2204 (43.5%)
Epidemiological history	0	0	0	0	0	0	0
Date of sampling	856 (98.1%)	880 (98%)	2552 (99%)	333 (99.7%)	329 (100%)	5 (100%)	4955 (98.7%)
Date of testing result	0	0	0	0	0	0	0
Current situation (treatment results for inpatient)	66 (84.6%)	119 (86.2%)	192 (86.9%)	28 (20.9%)	0	0	405 (63.5%)
Date of hospital discharge/referral/death (among inpatients)	65 (83.3%)	117 (84.8%)	188 (85.1%)	88 (65.7%)	34 (51.5%)	0	492 (77.1%)

**Table 4:** Comparison between previous paper-based surveillance system and eCDS.

Characteristics	Previous Paper-Based System	eCDS
Format	Paper-based	Computer-based
Responsible department	Department of General Planning	Department of Communicable Disease
Frequency	Monthly, quarterly, yearly	One time of reporting cases to the eCDS
Reporting data	Only number of infections and deaths	Specific information for each case includes demographics, clinical profiles, and reporting persons/unit.
Accessibility	Only higher level can have access data reported by lower levels.	Higher levels can still access data reported by lower levels. Lower levels (commune, district) can access data at provincial level.

## Data Availability

Data are not publicly available, due to privacy or ethical restrictions.

## References

[1] World Health Organization (2022). Hepatitis B. https://www.who.int/news-room/fact-sheets/detail/hepatitis-b.

[2] (2022). Global Health Sector Strategies on, Respectively, HIV, Viral Hepatitis and Sexually Transmitted Infections for the Period 2022–2030.

[3] (2022). Monitoring of Responses to the Hepatitis B and C Epidemics in EU/EEA Countries—2020 Data.

[4] Campbell C., Wang T., Smith D.A., Freeman O., Noble T., Várnai K.A., Harris S., Salih H., Roadknight G., Little S. (2021). Impact of the COVID-19 pandemic on routine surveillance for adults with chronic hepatitis B virus (HBV) infection in the UK. medRxiv.

[5] World Health Organization (2024). Global Hepatitis Report 2024: Action for Access in Low-and Middle-Income Countries.

[6] Ward J.W. (2021). Hepatitis B vaccines. Hepatitis B Virus and Liver Disease.

[7] World Health Organization (2017). Hepatitis B vaccines: WHO position paper–July 2017. Wkly. Epidemiol. Rec..

[8] Nguyen V.T. (2012). Hepatitis B infection in Vietnam: Current issues and future challenges. Asia Pac. J. Public Health.

[9] (2021). Decision, No. 4531/QD-BYT on Issuance of National Action Plan for Viral Hepatitis Prevention and Management 2021–2025.

[10] (2015). Circular 54/2015/TT-BYT Dated 28 December 2015 on Guidelines for Mechanism of Reporting and Declaration of Infectious Diseases and Outbreaks.

[11] German R.R., Horan J.M., Lee L.M., Milstein B., Pertowski C.A. (2001). Updated guidelines for evaluating public health surveillance systems; recommendations from the Guidelines Working Group. MMWR Recomm. Rep..

[12] (2022). Statistical Yearbook of Vietnam.

[13] (2023). Report of Population and Family Planning in Ninh Binh Province as of Quarter 1.

[14] (2023). Organization Chart of the Health Sector in Ninh Binh Province.

[15] (2019). Decision No. 3310/QD-BYT on Issuance of Guidelines for Diagnosis and Treatment of Hepatitis B.

[16] (2020). Completed Results of the 2019 Viet Nam Population and Housing Census.

[17] Boes L., Houareau C., Altmann D., An der Heiden M., Bremer V., Diercke M., Dudareva S., Neumeyer-Gromen A., Zimmermann R. (2020). Evaluation of the German surveillance system for hepatitis B regarding timeliness, data quality, and simplicity, from 2005 to 2014. Public Health.

[18] Tosti M.E., Longhi S., de Waure C., Mele A., Franco E., Ricciardi W., Filia A. (2015). Assessment of timeliness, representativeness and quality of data reported to Italy’s national integrated surveillance system for acute viral hepatitis (SEIEVA). Public Health.

[19] Cui F., Drobeniuc J., Hadler S.C., Hutin Y.J., Ma F., Wiersma S., Wang F., Wu J., Zheng H., Zhou L. (2013). Review of hepatitis B surveillance in China: Improving information to frame future directions in prevention and control. Vaccine.

[20] Kureta E., Basho M., Roshi E., Bino S., Simaku A., Burazeri G. (2016). Evaluation of the surveillance system for hepatitis B and C in Albania during 2013–2014. Albanian Med. J..

[21] Bonacic Marinovic A., Swaan C., van Steenbergen J., Kretzschmar M. (2015). Quantifying reporting timeliness to improve outbreak control. Emerg. Infect. Dis..

[22] Chung J., Yu J., Cheon M., Tak S. (2024). Evaluation of the acute hepatitis B surveillance system in the Republic of Korea following the transition to mandatory surveillance. Osong Public Health Res. Perspect..

[23] Kistan J. (2020). An Evaluation of the Performance of the Hepatitis B Surveillance System at Charlotte Maxeke Johannesburg Academic Hospital for 2017–2018. Ph.D. Thesis.

[24] Duffell E.F., van de Laar M.J., Amato-Gauci A.J. (2015). Enhanced surveillance of hepatitis B in the EU, 2006–2012. J. Viral Hepat..

[25] Mahmoodi M., Yazdanpanah A., Ghavam A., Sheikhzadeh K. (2019). Assessment of the core functions of hepatitis B surveillance system in the southeastern Iran: A qualitative study. Iran. J. Health Sci..

[26] (2024). Surveillance of Acute and Chronic Hepatitis B.

[27] (2016). Acute Hepatitis B: Enhanced Surveillance Questionnaire.

